# Contextualisation of Early and Enhanced Clinical Exposure Model through Development of Curricula for Advanced Practice Nursing and Midwifery

**DOI:** 10.4236/ojn.2022.127035

**Published:** 2022-07-29

**Authors:** Katowa-Mukwato Patricia, Mwiinga-Kalusopa Victoria, Maimbolwa Margaret Connie, Kabinga-Makukula Marjorie, Kayamba Violet, Kafumukache Elliot, Simuyemba Moses, Musenge Emmanuel, Mwiinga Christabel, Linda Kampata, Selestine H. Nzala, Cosmas Zyaambo, Trevor Kaile, Goma Fastone

**Affiliations:** 1 School of Nursing Sciences, University of Zambia, Lusaka, Zambia; 2 School of Medicine, University of Zambia, Lusaka, Zambia; 3 School of Public Health, University of Zambia, Lusaka, Zambia; 4 School of Medicine, South Valley University, Mazabuka, Zambia; 5 School of Medicine and Health Sciences, Eden University, Lusaka, Zambia

**Keywords:** Advanced Practice Nursing/Midwifery, Curricula Development, Early Enhanced Clinical Exposure, Model

## Abstract

**Background:**

An Advanced Practice Nurse is a generalist or specialized nurse who has acquired thorough graduate education a minimum of a master’s degree. The need for Advanced Practice Nurses is increasingly recognized globally. This paper describes the process, which was undertaken by School of Nursing Sciences, University of Zambia in reviewing and developing advanced practice nursing and midwifery curricula which will be implemented using the Early and Enhanced Clinical Exposure model (EECE).

**Materials and Methods:**

The curricula development/review process utilized a modified Taba’s Model which followed a step-by-step approach including: 1) desk review, 2) diagnosis of needs (needs assessment), 3) stakeholder consultations, 4) content development, 5) validations and approval from which several lessons were learnt and recommendations made. Findings and recommendations from different stages were used as a basis for reviewing and developing advanced practice nursing and midwifery curricula.

**Results:**

Desk review needs assessment and stakeholder consultations identified both strengths and weaknesses in the existing curricula. Major strengths were duration and core courses which met the minimum requirement for postgraduate nursing and midwifery training. Major weaknesses/gaps included some content that was too basic for the master’s level and the delayed exposure to practicum sites which limited the development of advanced practice skills. Others were inadequate competence for advanced practice, inadequate research methodology course, lack of content to foster development of personal soft skills and predominant use of traditional teaching methods. Stakeholders recommended implementing advanced, clinical and hands-on Masters of Nursing and Midwifery programmes which resulted in the review of four existing and development of five demand-driven curricula.

**Conclusion:**

The reviewed and developed curricula were strengthened to close the identified gaps. Both the reviewed and developed curricula have been implemented using the Early and Enhanced Clinical Exposure Model with a view to producing Advanced Practice Nurses and Midwives who are competent to meet diverse health care needs and contribute to improving patient outcomes.

## 1. Background

The need for Advanced Practice Nurses (APNs) with postgraduate education is increasingly recognized around the globe [1]. According to the International Council of Nursing (ICN), an Advanced Practice Nurse is a generalist or specialized nurse who has acquired thorough graduate education (minimum of a master’s Degree) with expert knowledge base, complex decision-making skills and clinical competencies shaped by the context in which they are credentialed [2] [3]. ICN [3] further identifies two most common APN roles as Clinical Nurse Specialist (CNS) and Nurse Practitioners (NP). CNS provides expert clinical advice and care based on established diagnoses in specialized clinical fields of practice as a member of the health care team, while NP integrates clinical skills associated with nursing and medicine in order to assess, diagnose and manage patients in Primary Health Care settings and acute care populations as well as ongoing care for populations with chronic illnesses.

Definition, recognition, legislation and regulation of ANP are at different stages of development in Europe and Northern America [3] For example, in the United States, the American Nurses Association (ANA) defined Clinical Nurse Specialists as registered nurses who have graduate-level nurse preparation at the master’s or doctoral level [4]. According to the American Nurses Association, CNSs practice autonomously and integrate knowledge of disease and medical treatment into assessment, diagnosis and treatment of patients’ illnesses [4]. They are experts in evidence-based nursing practice and practice within specialized areas in treating and managing health concerns of patients and populations.

In the United Kingdom (UK), the specialist nurse role was defined in the 1970s, to include a combination of clinical, education, research and consultancy. However, according to ICN 2020 [3], most CNS are not educated at the master’s level and the title CNS is not consistently used in all the four UK countries as there is no regulation around the use of the title. In New Zealand, the CNS role has no formal or legal definition, thus there is confusion about the CNS and the relevant scope of practice [5], however, the role of the CNS equates to the clinical nurse consultant in Australia [6] [7]. The qualification required for a CNS to practice in New Zealand varies according to the discretion of employers. In the Republic of Ireland, there is a framework that defines the CNS position [8]. CNS practice in New Ireland includes the provision of both direct care (assessment, planning, coordination, delivery and evaluation of care including patient and family education). On the other hand, the role of NP is well defined in Australia where the title is even protected and only nurses who have been authorized by the National Nursing and Midwifery Registration Board of Australia can use the title. NP roles include assessment, diagnosis, planning in collaboration with others, prescribing therapeutic interventions and evaluating care outcomes. While in Canada, NPs practice autonomously and can make diagnoses, order and interpret diagnostic tests, prescribe pharmaceuticals and perform specific procedures within their legislated scope of practice [9].

In Africa, Botswana is the only African institution with a master’s degree for the Family Nurse Practitioner that matches international Advanced Practice Nursing standards in education, accreditation and regulatory practice. According to Sibanda and Stender, five countries have set a priority to initiate Family Nurse Practitioner Programme and start working on Midwifery Advanced Practice [10]. This priority was set through a proposal to WHO (Africa) Health Systems Leadership under the Anglophone African Advanced Practice Nurse Coalition Project. This project may set a tone for developing streamlining and formalization of the Advanced Practice Nursing role in Africa. In 2018, Sibanda and Stender [10] further state that in Zambia, Kenya Malawi, Eswatini, South Africa, Uganda and Rwanda, the concept of APN has been identified, but the scope of practice and legislation to formalize their respective practices and legislation are not explicit.

In Zambia, the University of Zambia, School of Nursing Sciences (UNZA-SoN) has been training Postgraduate Nurses and Midwives since 2004. Initially, the School offered a single Master of Science in nursing programme from 2004 until 2015 when four distinct MSc Programmes were introduced: Master of Science in Clinical Nursing, Midwifery and Women’s Health, Mental Health Psychiatric Nursing, and Public Health Nursing. The programmes were for 2 years duration, with the first year dedicated to theoretical teaching and learning, while the first 6 months of the second year were dedicated to clinical practice and the last six months to research work and completion of the research dissertation. Using this model, the school graduated master’s degree level nurses and midwives who took up mostly teaching and administrative positions in different schools of nursing and care settings respectively. However, the scope of practice and legislation for such nurses and midwives has not been formalized.

Having administered the specific master’s degree curricula for five years from 2015 to 2020, the programmes were due for review at the end of the 2019 academic year. This paper provides a narrative of the processes that culminated into the review and development and subsequent implementation of Advanced Practice nursing and midwifery Programmes at the University of Zambia School of Nursing Sciences. The existing curricula which were reviewed are Clinical Nursing, Midwifery, Public Health and Mental Health and Psychiatric Nursing, while the new need-based programmes which were developed based on the needs assessment and recommended from stakeholder consultations included MSc Palliative Care, Oncology, Neonatology, Critical Care and Trauma and Emergency Nursing. In order to facilitate the transition from the previous curricula and model of administering masters’ programmes, UNZA-SoN conceived a model which was referred to as Early and Enhanced Clinical Exposure Model (EECE). EECE is a novel model which entails immediate placement of nursing and midwifery masters students and their integration into the clinical area for continued hands-on learning throughout the training period. This paper reports the process of curricular review and implementation and the outcomes of that process together with the potential benefits and challenges of the proposed model.

## 2. Materials and Methods

As an educational and quality assurance requirement, in 2020, UNZA-SoN undertook a review of its existing MSc programmes. The curricula development/review process utilized a modified Taba’s Model [11] (which followed a step by step approach including, 1) desk review, 2) diagnosis of needs (needs assessment), 3) stakeholder consultations, 4) content development, 5) validations and approval from which several lessons were learnt and recommendations made. The needs assessment involved employers, regulatory and professional bodies, current students and former graduates. Findings and recommendations from different stage were used as a basis for reviewing and developing advanced practice nursing and midwifery curricula (Table 1).

## 3. Results

Findings of the different phases of the curricula review and development are outlined in Table 2. Whereas the desk review of the existing curricula identified mainly strengths and weaknesses, the needs assessment and stakeholder consultation findings were mainly in form of suggestions for advanced nursing programmes which stakeholders required to be implemented. The rest of the phases are inherent curricula review/development stages and approvals.

## 4. Discussion of Findings

With stakeholder recommendations for review and development of new MSc programmes, the SONs reviewed four existing masters curricula and developed five new programmes in order to train nurses and midwives with advanced practice competencies. The aspiration of the Zambia stakeholder for the country to have nurses and midwives trained at advanced practice level was in line with ICN, 2020 release of new guidelines on advance practice nursing, and its subsequent declaration that APNs are an effective and efficient resource to address the challenges of accessible, safe and affordable health care. According to literature, Master’s programs in Nursing Science curricula prepare graduates for Advanced Practice roles [12]. With the newly developed and reviewed masters programmes having a significant part of training dedicated to clinical practice, it is hoped that graduates will be equipped with necessary skills for meeting diverse health care needs. In order to meet the increasing demands of modern healthcare, nurses are required to possess advanced and specialized expertise which are acquired with advanced or masters education training [13].

In order to equip graduates with appropriate knowledge to support advanced practice, the reviewed and developed masters curriculum had to incorporate key concepts such as evidence based practice, critical thinking, collaborative practice, nursing informatics and research skills which are required for provision of advanced nursing and midwifery care. Possession of such competences is important as in the modern clinical setting where nurses are challenged to think critically and make autonomous decisions as such modern nurses increasingly need advanced nursing education to succeed in their clinical role [13]. Benefits of having masters’ educated nurses in the clinical setting are evident in literature and have been document by a number of scholars and thus the aspiration for each country to have nurses and midwives trained at that level. Such benefits, include improved health care accessibility, chronic care management, health care outcomes, and reduced waiting time and average hospital stays [14] [15] [16]. While benefits related to women, reproductive health, gynaecology and ob stetrics include reduced new born mortality and increased likelihood of breast feeding [17].

In order to facilitate the transition from the previous curricula and model of administering masters’ programmes, UNZA-SoN conceived a framework which was referred to an Early and Enhanced Clinical Exposure Model **(**Appendix 1). The framework has three facets 1) Early Clinical Exposure 2) enhanced clinical exposure and 3) Integration of students within the clinical time table. Early clinical exposure entails that upon commencement of training, students will be in class for introductory lesson for 6 weeks thereafter, they will be placed in the discipline related clinical settings where three days in a week will be spent in the clinical sites, theoretical learning will be conducted for one day per week and one day dedicated to self-reflection (Appendix 1). Meanwhile Enhanced Clinical Exposure entails 1:3 Theory-to-Clinical Teaching Ratio. Students will spend three quarters (3/4) of their training time in the clinical area and a quarter (1/4) for didactic or theoretical teaching/learning, Meanwhile integration of students within the clinical time table means that Postgraduate Nursing and Midwifery students will be part of the clinical time table within the specific placement areas except for those hours that they will be required to be in class to enhance acquisition of key cognitive related competencies.

### 4.1. Potential Benefits of the Early and Enhanced Clinical Exposure Model

The devised model of training has a number of potential benefits. Postgraduate Nursing and Midwifery trainees will be expected to learn from both the few available nurses and midwives with masters’ level qualification as well as from the Physicians, Surgeons, Paediatricians and Obstetricians depending on the programme of training. It is envisioned that this model of training will result in subsequent graduation of Masters level Nurses and Midwifery Practitioners who will be hands-on-and more adaptable to work in a multidisciplinary clinical setting rather than focusing on teaching jobs as has been the case before. As asserted by Wangensteen *et al*. and Cotterill-Walker [18] [19], a nurse who is educated to master’s degree level is better equipped for analytical thinking and interdisciplinary collaboration. In addition, Cotterill-Walker argues that the benefits of a master’s qualification in improving Registered Nurses’ capacity to understand and use medical terminology and to practice critical thinking, which facilitates communication with doctors and other professionals [19]. This will in the long term promote team work and interprofessional practice which has potential for improving patient outcomes. It is assumed that early and enhanced clinical exposure will enable the Postgraduate Nurses and Midwives to identify and conduct research within the clinical areas which will allow for generation of evidence-based knowledge from the local context thereby solving local nursing and midwifery problems and contributing to improving the quality of care through Evidence Based Practice. This assumption is supported by others scholars such as Gerrish 2003, who indicated that evidence-based practice can facili tate the development of new improved solutions and increase the health service’s credibility. In addition, Drennan [20] concluded that taking a master’s degree significantly improves nurses’ knowledge, making them better equipped to change practice, communicate, contribute to teamwork, resolve problems and address challenges.

Masters students are mainly senior and experienced nurses and midwives with basic diploma and or bachelor’s degree qualifications in Registered Nursing, Operating Theatre Nursing, Critical Care, Paediatric Nursing, Oncology Nursing and Midwifery. Having such masters students integrated within the clinical area will help enhance provision of the much needed quality nursing and midwifery care from the experienced nurses and midwives. Nurses with master’s degrees can make a positive contribution to the quality of services and patient safety [21]. Being first degree holder nurses and midwives, some master’s students have vast practice experience, as such their presence will provide the much needed mentorship to junior nurses and midwives. This aspect of mentorship has been lost over the recent years in the Zambia context. In addition, Postgraduate Nurses and Midwifery trainees will act as preceptors for skills transfer to other low level nursing/midwifery students within the different clinical placement areas.

### 4.2. Potential Challenges of the Early and Enhanced Clinical Exposure Model

One major challenge is there are currently very few nurses and midwives in the clinical settings who are qualified at master’s level to support implementation of advanced practice nursing and midwifery. To achieve the proposed model, it will require support of other clinicians mainly Medical Doctors of which this support cannot be guaranteed at all times.

## 5. Conclusion

In line with stakeholders’ recommendations to implement advanced clinical based hands-on Masters of Nursing and Midwifery programmes, UNZA-SoN reviewed and developed Advanced Practice Nursing and Midwifery curricula, and has positioned itself to implement the “Early and Enhanced Clinical Exposure Framework” for all its MSc Programmes with a view to producing Advanced Practice Nurses and Midwives. The stakeholders’ recommendations are supported by the need for improved nursing and midwifery care, and the need for mentorship and guidance for junior nurses, midwives and undergraduate students, which can all be achieved through the proposed model of training. It is assumed that having nurses and midwives trained at the advanced practice level may accrue benefits that have been documented by several scholars including improved health care accessibility, chronic care management, health care outcomes, and reduced waiting time and average hospital stay. One challenge though remains for the Nursing and Midwifery profession in Zambia is to define the scope of practice and formalize legislation for advanced practice nursing and midwifery in Zambia.

## Figures and Tables

**Table 1.** Phases of the curricula review/development process.

Phase/Activity	Description
Phase 1: Desk Review	Desk review of curricula and professional documents on nursing and midwifery higher education at national, regional and international levels was undertaken to identify trends and strategic directions for nurse/midwifery education
Phase 2: Needs Assessment	A needs assessment was undertaken through a survey questionnaire administered to representatives of; 1) The major employer for nurses and midwives-the Ministry of Health 2) United Nations Organizations including World Health Organization, country office and United Nations Population Fund (UNFPA) 3) Professional bodies including Nursing and Midwifery Council, Zambia 4) Professional Associations-the Midwifery Association of Zambia, the Zambia Union of Nurses Organizations and Palliative Care Association of Zambia 5) Deans and Principals of Health Professions Education Institutions 6)Nursing and Midwifery Graduates 7) Nursing and Midwifery Students 8) Patient Associations-Breast Cancer Survivors 9) Community representations
Phase 3: Stakeholder Consultation	A consultative Stakeholders meeting was held with representatives from the Ministry of Health (MOH) Nursing & Midwifery Council of Zambia, Professional bodies e.g. Eastern, Central and Sothern African College of Nursing (ECSACON) & Zambia Union of Nurses Organization, School of Medicine Medical Doctors’ Association, BSc Nurse Alumni and UNZA Nursing Students’ Union
Phase 4: SWOT Analysis	The School curriculum committee conducted a SWOT analysis to analyze the Strengths, Weaknesses, Opportunities and Threats was conducted to help the school build on where it was doing well, address what was lacking to minimize risks and take advantage of opportunities for success
Phase 5: Curricula Review/Development	Based on the findings of the needs assessment, desk review, stakeholder consultations, existing curricula were reviewed while new curricular were developed taking into considerations the recommendations and findings from the bench-marking process. To facilitate achievement of early and enhanced clinical exposure, a curricular implementation framework was developed indicating the different educational activities for the two year training duration
Phase 6: School Board Approval and University Senate Approval	The School Board and University Senate which are composed of different stakeholders, from university administrators, nursing and midwifery professionals, educational experts, community members and students representatives reviewed and approve the curricula prior to implementation
Phase 7: Curricula submission to the Nursing and Midwifery Council of Zambia (NMCZ)	The four reviewed and five developed Master of Science in Nursing and Midwifery curricula were submitted to the Nursing and Midwifery Council for review, registration and recognition of qualification by the Nursing and Midwifery regulatory body in the Country
Phase 8: Submission to the High Education Authority	The four reviewed and five developed Master of Science in Nursing and Midwifery curricula were submitted to the Higher Education Authority for Accreditation

**Table 2.** Findings/outcomes of the curricula review/development process and approvals.

Activity	Findings/Outcomes
Phase 1: Desk Review	1) Strengths and weakness of existing MSc Nursing and Midwifery curricula; a) Duration of training and core courses met minimum standards for masters of science in nursing and midwifery b) Some content were too basic for masters level, which limited development of advanced practice skills c) Competences for evidence based practice, critical thinking and implementation science were missing d) Research methodology course was inadequate with regards to academic writing, literature search and reviews, qualitative methodologies and data analysis e) Lack of standard guide for proposal and dissertation writing f) Lack of content to foster development of personal soft survival skills including entrepreneurship, quality improvement, collaborative practice, nursing informatics and technology, self-management, team building and transformational leadership g) Predominant use of traditional teaching methods. h) Delayed exposure to practicum sites 2) Most regional and international masters nursing curricula are of two years duration 3) Curricula for Masters of Nursing are broadly guided by the ICN 2020 guidelines on advanced practice nursing and WHO (2005) guidelines for preparing a health care workforce for the 21st century
Phase 2: Needs Assessment	a) There was greater need for more specialized nursing and midwifery programmes to increase the number of graduates to provide advance practice care in hospitals including primary care settings b) Fourteen masters programs were identified to be developed by the school (Oncology Nursing, Palliative care Nursing, Infectious diseases and HIV nurse practitioner, Neonatology Nursing, Critical care Nursing, Nephrology Nursing, Trauma and emergency nursing, Child and adolescent psychiatry) c) Out of the 14, five were prioritized and recommended for immediate development (Critical Care Nursing, Emergency and Trauma Nursing, Neonatal Nursing, Oncology Nursing, and Palliative Care) d) The assessment methods should be tailor-made to each programme to ensure the core objective of saving life & preventing permanent disabilities e) Challenges highlighted—included inadequate specialized staff, inadequate infrastructure especially the skills laboratory, financial constraints f) Solutions to identified challenges included establishment of fellowships to promote capacity building, signing of an MoU with the University Teaching Hospital & other hospitals so that students can begin practice early, equip the clinical skills laboratory with more equipment and simulators and , promoting online teaching
Phase:3 Stakeholder Consultation	1) Stakeholders expressed willing to support UNZA-SoN in curricula review/development and subsequent programme implementation 2) Recommended to: a) Review and develop curricula that addresses gaps identified during the desk review and needs assessment b) Introduce early and enhanced clinical exposure c) Identifying clinical mentors from the practicum sites to facilitate mentorship and enhance clinical competencies of students and graduates d) Introduce students to some high level clinical skills e.g. conducting lumbar puncture, incision & drainage, intubation in order to produce nurse and midwifery practitioners who are hands-on, more practical and service delivery oriented
Phase 4: SWOT Analysis	1) Strengths; UNZA-SoN rated as number one among universities in Zambia, has established MSc implementation systems, offers different modes of study 2) Weaknesses; limited infrastructure, intermittent internet connectivity, inadequate specialized staff 3) Opportunities; UNZA-SoN is a recognized institution globally and rated as number 1 in Zambia, strong partnerships with global and regional institutions, availability of different platforms for online teaching and good will from stakeholders 4) Threats: competition from up-coming universities
Phase 5: Curricula Review/Development	1) Four master of sciences curricula reviewed; Clinical Nursing, Midwifery and Women’s Health, Public Health Nursing and Mental Health Nursing 2) Five masters of science curricula developed; Critical Care Nursing, Emergency and Trauma Nursing, Neonatal Nursing, Oncology Nursing, and Palliative Care
Phase 6: School Board Approval and University Senate Approval	1) Four reviewed curricula approved as academic masters programmes 2) Four New curricula approved as academic Masters 3) One (Critical Care Nursing) approved as Professional Masters Programme
Phase 7: Curricula submission to the Nursing and Midwifery Council of Zambia (NMCZ)	The four reviewed and five developed Master of Science in Nursing and Midwifery curricula registered by the Nursing and Midwifery Council of Zambia
Phase 8: High Education Authority (HEA) Approval and Accreditation	The four reviewed and five developed Master of Science in Nursing and Midwifery curricula accredited by Higher Education Authority
